# Eyes-Closed Resting EEG Predicts the Learning of Alpha Down-Regulation in Neurofeedback Training

**DOI:** 10.3389/fpsyg.2018.01607

**Published:** 2018-08-28

**Authors:** Wenya Nan, Feng Wan, Qi Tang, Chi Man Wong, Boyu Wang, Agostinho Rosa

**Affiliations:** ^1^Department of Psychology, Shanghai Normal University, Shanghai, China; ^2^Department of Electrical and Computer Engineering, Faculty of Science and Technology, University of Macau, Macau, China; ^3^Princeton Neuroscience Institute, Princeton University, Princeton, NJ, United States; ^4^Department of Bioengineering, LaSEEB-System and Robotics Institute, Instituto Superior Tecnico, University of Lisbon, Lisbon, Portugal

**Keywords:** neurofeedback, alpha, down-regulation, resting baseline, learning

## Abstract

Neurofeedback training, which enables the trainee to learn self-control of the EEG activity of interest based on online feedback, has demonstrated benefits on cognitive and behavioral performance. Nevertheless, as a core mechanism of neurofeedback, learning of EEG regulation (i.e., EEG learning) has not been well understood. Moreover, a substantial number of non-learners who fail to achieve successful EEG learning have often been reported. This study investigated the EEG learning in alpha down-regulation neurofeedback, aiming to better understand the alpha learning and to early predict learner/non-learner. Twenty-nine participants received neurofeedback training to down-regulate alpha in two days, while eight of them were identified as non-learners who failed to reduce their alpha within sessions. Through a stepwise linear discriminant analysis, a prediction model was built based on participant’s eyes-closed resting EEG activities in broad frequency bands including lower alpha, theta, sigma and beta 1 measured before training, which was validated in predicting learners/non-learners. The findings would assist in the early identification of the individuals who would not likely reduce their alpha during neurofeedback.

## Introduction

Neurofeedback (NF) training is a type of brain training where neural activity of interest is measured and fedback to the trainee in real time by visual, auditory, or visual-auditory representation in order to facilitate self-regulation of the putative neural substrates that underlie a specific behavior or pathology (Sitaram et al., 2017). Over the years, an increasing number of studies have utilized NF training as a non-invasive tool to enhance cognition, affection and creativity in healthy people (Gruzelier, 2014a,b; Enriquez-Geppert et al., 2017), to normalize patients’ abnormal brain activity for treatment of symptoms in brain disorders such as attention deficit hyperactivity disorder (ADHD) and stroke rehabilitation (Sitaram et al., 2017), to improve brain-computer interface (BCI) performance (Wan et al., 2016; McWhinney et al., 2017), as well as to investigate the causality between neural activity and cognition/behavior (Enriquez-Geppert et al., 2017).

In NF training, learning of EEG self-regulation (or called EEG learning) is the core mechanism and a marker of specific NF effects (Baumeister et al., 2016). Specifically, it has been found that EEG learning is closely related to the improvement of performance after training in a variety of NF protocols (Egner and Gruzelier, 2003; Ros et al., 2009; Keizer et al., 2010; Nan et al., 2012; Gruzelier, 2014a), and it influences the transfer effects of NF from laboratory to real-world conditions (Sitaram et al., 2017). Although many trainees can gain successful learning to regulate the brain activity of interest in a desired direction, a substantial portion of subjects have been reported unsuccessful in EEG learning, regardless of the NF protocol and subject population (Lubar et al., 1995; Kotchoubey et al., 1999; Hanslmayr et al., 2005; Kropotov et al., 2005; Doehnert et al., 2008; Weber et al., 2011; Zoefel et al., 2011; Kouijzer et al., 2013; Enriquez-Geppert et al., 2014; Schabus et al., 2014; Reichert et al., 2015; Baumeister et al., 2016; Hsueh et al., 2016; Quaedflieg et al., 2016). The rates of this type of so-called non-learners vary, up to around 50% in some studies (Hanslmayr et al., 2005; Doehnert et al., 2008; Okumura et al., 2017). What is more, the non-learners often show less improvement on behavior/symptoms than learners (Lubar et al., 1995; Kropotov et al., 2005) or even no improvement after NF training (Hanslmayr et al., 2005; Kouijzer et al., 2013; Hsueh et al., 2016), which seriously affects the efficacy of NF training.

Some researchers attempted to explore the individual difference of EEG learning from different aspects (Gruzelier, 2014c; Zuberer et al., 2015; Sitaram et al., 2017). By a computation-theoretic approach, Davelaar (2017) showed a critical role of the striatum in learning to up-regulate alpha activity. Additionally, several studies investigated whether and how the psychological factors such as mental strategies, control beliefs, concentration, mood, locus of control and motivation influence the learning in different NF protocols (Gruzelier et al., 1999; Nijboer et al., 2010; Nan et al., 2012; Kober et al., 2013; Witte et al., 2013; Enriquez-Geppert et al., 2014; Sitaram et al., 2017).

Some other research efforts have been paid to identifying neurophysiological predictors for EEG learning, which can assist in predicting the EEG learning, avoiding time consuming sessions on non-learners and understanding the underlying mechanism of individual difference in EEG learning. For instance, in sensorimotor rhythm (SMR) learning, predictors were the SMR power in the middle process of training (Weber et al., 2011), the SMR power at resting baseline before NF (Reichert et al., 2015), and the different brain structural properties (Ninaus et al., 2015). Similarly, theta learning was reported predictable by the brain structural properties (Enriquez-Geppert et al., 2013), while beta/theta ratio learning could be predicted by resting and initial training beta activity (Nan et al., 2015).

For the prediction of alpha learning, our prior work found that the learning of alpha up-regulation was related to the resting alpha amplitude in both eyes-open and eyes-closed resting condition (Wan et al., 2014). Contrary to up-regulation, in down-regulation protocol that could induce cortical activation (Ros et al., 2010, 2013), the investigation of alpha learning is rare. Although some studies reported that alpha amplitude was smaller in one 30-min session when compared with resting baseline (Ros et al., 2013) or it reduced across two 15-min NF sessions (Wan et al., 2016), the within-session learning that describes alpha dynamic change within sessions is not clear yet. Moreover, previous work mainly reported the alpha learning at the group level whereas the inter-individual difference in alpha learning was little known (Ros et al., 2010, 2013, 2014b, 2016; Wan et al., 2016).

In summary, learning down-regulation of alpha through NF training has not been well understood yet, especially the within-session learning, its individual difference and predictors. Therefore, this study investigated the learning of alpha down-regulation for better understanding the learning process and identifying the neurophysiological predictors of learner/non-learner. Twenty-nine participants performed NF training to learn down-regulation of their alpha activity in two consecutive days through real-time visual feedback. Inspired by the importance of resting EEG activities in prediction of EEG learning in other protocols (Wan et al., 2014; Nan et al., 2015; Reichert et al., 2015), we focused on the resting EEG activities measured before NF to predict learner and non-learner in alpha down-regulation NF training.

## Materials and Methods

### Participants

A total of 29 healthy volunteers (9 females) aged from 22 to 32 years participated in the experiment. All the participants had no history of psychiatric or neurological disorders, no psychotropic medications or addiction drugs, and with normal or corrected-to normal vision. They signed an informed consent form before experiment and received monetary compensation for their participation after experiment. The protocol was in accordance with the Declaration of Helsinki and approved by the local Research Ethics Committee (University of Macau).

### Experiment

Since alpha down-regulation NF training at Oz showed positive effects on BCI performance enhancement in Wan et al. (2016), this study utilized the same training location. EEG signal at Oz channel was acquired by an amplifier of g.USBamp (Guger Technologies, Graz, Austria) with a sampling rate of 256 Hz. The ground electrode was placed on the forehead and the reference location was the left mastoid. The impedance was kept below 10 kΩ.

Each participant performed one training session per day in two consecutive days. Each session consisted of five blocks, and each block had three 1-min trials with an interval of 5 s between two consecutive trials. Resting EEG signals were recorded before and after training, denoted as Baseline 1 and Baseline 2, respectively. Each baseline consisted of four 30-s epochs with eyes open and four 30-s epochs with eyes closed.

Since large individual differences have been found in the alpha frequency (Klimesch, 1999), the NF training focused on the individualized alpha frequency band based on the peak alpha frequency (PAF) that was the frequency with the largest amplitude located within 7.5 to 12.5 Hz in the eyes-closed Baseline 1 (Klimesch, 1999). The alpha frequency band ranged from low transition frequency (LTF: PAF-2 Hz) to high transition frequency (HTF: PAF+2 Hz) (Dekker et al., 2014). For real-time feedback, a band-pass filter (0.5–30 Hz) was applied to avoid the high frequency noise, baseline drift as well as powerline interference.

The training parameter was the relative alpha amplitude calculated from equation (1), where *X*(k) was the frequency spectrum amplitude calculated by fast Fourier transformation (FFT) with a 1-s sliding window that shifted forward every 0.125 s, Δ*f* was the frequency resolution of FFT and *k* was the spectrum index (Wan et al., 2016).

$$\text{Relative alpha amplitude=}\frac{\overset{HTF/\Delta f}{\underset{k=LTF/\Delta f}{\Sigma }}X\left(k\right)}{HTF-LTF}/\frac{\overset{30/\Delta f}{\underset{k=0.5/\Delta f}{\Sigma }}X\left(k\right)}{30-0.5}$$

A sphere and a cube displayed on a computer screen were utilized for real-time visual feedback. The radius of the sphere reflected a real-time feedback of the training parameter. If the training parameter was below a pre-defined threshold (Goal 1), the sphere changed its color from white to purple, and its size increased as the training parameter decreased. The cube height increased whenever the feedback parameter stayed below the threshold for more than 2 s (Goal 2). Thus, participants were asked to perform spontaneous mental thoughts in order to achieve the goals for their alpha reduction (Wan et al., 2016).

According to our experimental experience, the threshold in the first training block was set to equal to or slight higher than the resting alpha in the eyes-open Baseline 1. For the remaining blocks, the threshold would be decreased by 0.1 if the percent time below threshold was above 60%.

### EEG Amplitude Offline Calculation

Considering that the absolute EEG amplitude is easily influenced by many factors such as anatomical and neurophysiological properties of the brain, cranial bone structure and electrode impedances (Kropotov, 2010), relative EEG amplitude (relative to 0.5–30 Hz, EEG amplitude for short) was calculated in order to ensure comparability across participants and across training time (Reichert et al., 2015). For both eyes-open and eyes-closed resting baseline before and after NF, the EEG amplitude was computed in not only alpha but also theta (4–8 Hz), lower alpha (PAF-2 to PAF), upper alpha (PAF to PAF+2), sigma (12–16 Hz), beta 1 (16–20 Hz), and beta 2 (20–28 Hz) frequency bands. During NF training, the alpha amplitude in each 1-min trial was calculated, and then the averaged alpha in three trials was taken as block activity for following analyses.

### Alpha Learning Assessment

The alpha learning was evaluated within sessions. To quantify within-session learning for each participant, the mean alpha change of Block 2 to Block 5 compared to Block 1 within one session averaged over two sessions was taken as the learning index, which described the average learning ability in short term (Wan et al., 2014). As the training objective was to decrease alpha over training time, the participant with negative learning index was defined as learner and the participant with positive learning index was defined as non-learner.

### Statistical Analysis

The following statistical analysis was conducted by SPSS 22 Software. Firstly, paired *t* test was employed to examine the amplitude difference between Baseline 1 and Baseline 2 in each frequency band. Furthermore, repeated-measures ANOVA with Block (5 levels: Block 1 to Block 5) and Session (2 levels: Session 1 and Session 2) as within-subjects factors was performed on alpha amplitude to examine alpha change during training. Greenhouse-Geisser adjustments were used if Mauchley’s test showed violations of the sphericity assumption.

Two-tailed Pearson correlation test was applied to examine the relationship between the learning index and EEG features in Baseline 1. Furthermore, in order to predict learners and non-learners, we employed a stepwise linear discriminant analysis (LDA) that is widely applied in the class prediction (Kleih and Kubler, 2013; Reichert et al., 2015). This method consists of two process stages. Firstly, the useful features are selected from all input variables by a stepwise process based on their effects on the separation between the two groups. Here, all of the input variables were the amplitudes in all frequency bands in Baseline 1. Secondly, the coefficients of selected feature variables are determined in the discriminant function to achieve maximum separation of two groups (Chan et al., 1995). As a result, a discriminant function is formulated as a linear combination of the useful feature variables. As shown in the equation 2, *n* is the number of useful feature variables *X*_i_, whose coefficients *a*_i_ are calculated in order to achieve a maximum separation between the distributions of the discriminant scores, D, of the two groups (Chan et al., 1995).

$$D=a_{0}+\overset{n}{\underset{i=1}{\Sigma }}a_{i}X_{i}$$

## Results

### Resting Baseline Change

Neither eyes-open nor eyes-closed resting alpha amplitude showed significant difference between Baseline 1 and Baseline 2 (all *p* > 0.05). Likewise, the amplitudes in other frequency bands including theta, lower alpha, upper alpha, sigma, beta 1, and beta 2 had no significant difference between Baseline 1 and Baseline 2 in the eyes-closed resting state (all *p* > 0.05). But it was not the case for the eyes-open resting baseline. Theta amplitude showed significant reduction [*t*(28) = -2.15, *p* = 0.04] and beta 1 amplitude had significant enhancement [*t*(28) = 2.271, *p* = 0.031] in the eyes-open Baseline 2 compared to Baseline 1.

### Alpha Change in NF Training

**FIGURE 1:**
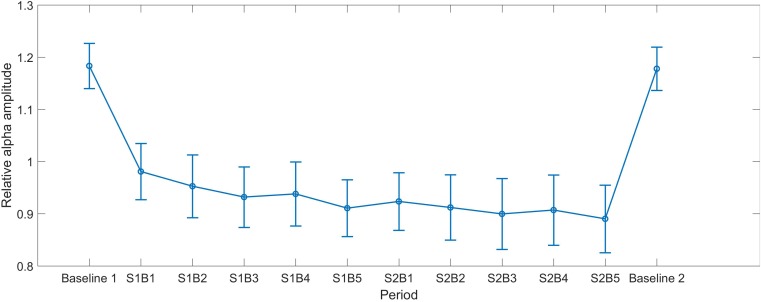
The mean alpha curves across all periods. S indicates session and B indicates block, e.g., S1B1 indicates the first block in Session 1. Baseline 1 and Baseline 2 represent the eyes-open baseline before and after NF, respectively. The error bars represent the standard error of the mean (SEM).

As shown in **Figure 1**, the alpha amplitude had a decrease trend over training periods. Repeated ANOVA revealed a significant main effect of Session [*F*(1,28) = 4.446, *p* = 0.044, partial η^2^ = 0.137], indicating that alpha in Session 2 (*M* = 0.907, *SEM* = 0.062) was smaller than that in Session 1 (*M* = 0.943, *SEM* = 0.056). The main effect of Block was marginally significant [*F*(2.864,112) = 2.602, *p* = 0.06, partial η^2^ = 0.085], whereas no interaction between Session and Block was identified [*F*(4,112) = 0.654, *p* = 0.625, partial η^2^ = 0.023].

### Learner and Non-learner

At the individual level, the alpha learning index ranged from -0.78 to 1.27. The alpha learning index had a positive correlation with the amplitude in the alpha band in the eyes-closed Baseline 1 (*r* = 0.394, *p* = 0.034). In addition, the alpha learning index was also positively related to the amplitude in the lower alpha band in both eyes-closed (*r* = 0.508, *p* = 0.005) and eyes-open (*r* = 0.476, *p* = 0.009) Baseline 1. Twenty-one participants with negative learning index were split into the learner group, whereas eight participants with positive learning index were split into the non-learner group.

**FIGURE 2:**
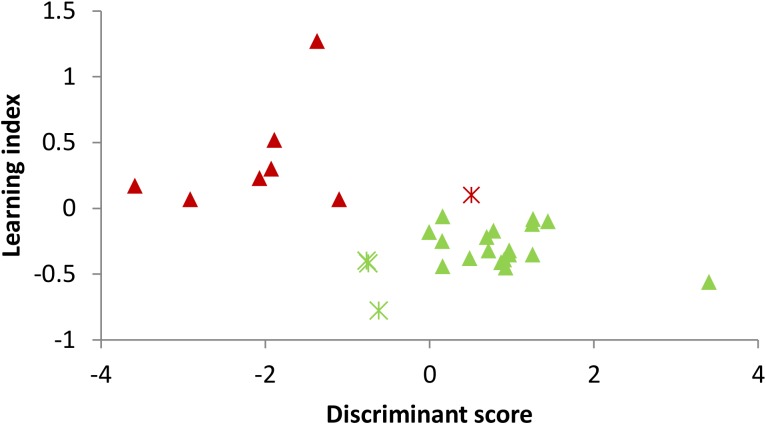
The participants’ discriminant scores and the prediction results of learners and non-learners. Each dot represents the discriminant score of each participant. Green triangle: learner with correct prediction; Green asterisk: learner with wrong prediction; Red triangle: non-learner with correct prediction; and Red asterisk: non-learner with wrong prediction.

In order to predict the learners and non-learners, the stepwise LDA was applied to extract useful features and build prediction model. The results showed that the amplitudes in the lower alpha, theta, sigma and beta 1 frequency bands together in the eyes-closed Baseline 1 were the significant predictor variables of learners and non-learners, with a 86.2% leave-one-out cross-validation accuracy. More specifically, the discriminant score of each participant is calculated by the discriminant function formed by a linear combination of these EEG predictor variables. In prediction of EEG learning of in total 29 participants, 7 non-learners and 18 learners were successfully predicted with the resulting prediction model. For visualization, **Figure 2** depicts the prediction result, in which each dot represents one participant, whose horizontal coordinate is the participant’s calculated discriminant score, and the vertical coordinate is the learning index. The triangles and asterisks in green denote learners and the red triangles and asterisks stand for the non-learner. It can be seen that the found discriminant function could separate learner and non-learner groups with an acceptable accuracy.

## Discussion

Considering the importance of EEG learning and the non-learner problem, this study aimed to investigate the learning of alpha down-regulation through NF training. Twenty-nine participants completed NF training in two consecutive days. In accordance with the training goal, most of the participants learnt to down-regulate their alpha amplitude. Nevertheless, 8 non-learners occurred, accounting for 27.59% of total participants. The non-learner phenomenon has been reported in a variety of NF protocols, and the non-learner rate in this study is consistent with previous alpha NF studies showing that 20–50% of participants are not able to regulate their alpha activity by NF training (Hanslmayr et al., 2005; Zoefel et al., 2011). Remarkably, we demonstrated that eyes-closed EEG amplitudes in broad frequency bands including lower alpha, theta, sigma and beta 1 measured before NF could be used to predict learners and non-learners.

For the resting alpha amplitude, neither eyes-open nor eyes-closed alpha showed significant difference between Baseline 1 and Baseline 2. This result coincided with alpha down-regulation NF from Ros et al. (2013) in which the alpha in the first training run with three min in healthy participants was significant lower than that in the pre-baseline, and the alpha in the post-baseline after 30 min training rebounded to pre-baseline level. No significant change in resting alpha may be attributed to short NF duration or small session number since only one NF session with about 30 min was performed in previous studies (Ros et al., 2013, 2016; Alves-Pinto et al., 2017) and only two 15-min sessions were conducted in this work. However, multiple NF sessions with much longer training duration, such as six NF sessions with 20 min each, also showed no significant changes in resting alpha amplitude (Ros et al., 2017). To sum up, despite the down-regulation direction, the resting alpha amplitude was hard to change in this type of training, which may be independent of session number. As pointed out by Ros et al. (2014a), NF tunes EEG oscillations toward a homeostatic set-point, affording an optimal balance between network flexibility and stability. Nevertheless, it has been found that stronger “rebound” in alpha is associated with the greater alpha reduction during NF (Nicholson et al., 2016) (i.e., good alpha learning).

The assessment of EEG learning is diverse in the literature. In general, EEG learning is usually evaluated by the training parameter change within sessions, across sessions, or within sessions compared to baselines (Gruzelier, 2014c; Wan et al., 2014; Nan et al., 2015; Reichert et al., 2015; Zuberer et al., 2015). With respect to alpha down-regulation NF, although Ros and colleagues did not directly use the term “alpha learning” or “NF learning,” they reported the comparison of alpha amplitude between NF session and resting baseline (Ros et al., 2010, 2013, 2016), which was actually one type of alpha learning assessment. Since it has been observed that alpha also shows reduction in sham NF group compared to resting baseline (Ros et al., 2013) whereas this study did not include sham NF group for comparison, the alpha change between NF session and resting baseline may increase the difficulty to identify real NF effects on alpha. What is more, the sustained alpha reduction during NF was the objective of NF training. Thus, we paid more attention on the alpha change during NF training. More specifically, the alpha learning was only examined from alpha change within sessions rather than across sessions due to the following considerations. Firstly, within-session learning calculated based on the training parameter within one session averaged over multiple sessions may smooth the overall sampling error variance and result in a more robust indicator of learning dynamics (Gruzelier et al., 2014). Secondly, the session number in this study was relatively small. Zuberer et al. (2015) argued that only a small number of single sessions for the calculation of across-session learning is often problematic since some external variables unrelated to the training such as day-to-day events, fluctuating arousal levels and sleep patterns may lead to biased performance of a single session. Thirdly, it has been suggested that focusing on within sessions changes may be a more useful approach in identifying alpha changes resulting from NF training (Dempster and Vernon, 2009). Therefore, this study focused on the within-session learning only.

Overall, the significant main effect of Session and the marginal significant effect of Block indicated that the participants could learn to down-regulate their alpha amplitude over training periods. From this point of view, we can say that the training was successful. However, at the individual level, large individual difference in learning was presented. Contrary to the training goal, 27.59 % of participants failed to show alpha reduction within sessions, which was similar with the sham group performance from Ros et al. (2013) in which the feedback signal was from a NF-successful participant rather than the trainee’s own EEG activity.

To our knowledge, the quantitative analysis of alpha learning at the individual level is rare in alpha down-regulation NF. Ros et al. (2017) reported the individual alpha learning under six 21-min NF sessions. In the first session, only one participant successfully learnt down-regulation of alpha in the seven training runs compared to pre baseline. This further confirmed the large individual difference in learning of alpha down-regulation by NF training.

A moderately positive correlation was found between alpha learning index and resting alpha activity, which implies that an individual with lower resting alpha activity would have a relatively lower learning index value or say relatively better learning in alpha down-regulation. This is interesting when comparing with the result from Wan et al. (2014) where higher resting alpha activity was related to better learning in alpha up-regulation. Taken both studies together, we speculate that the participants with larger alpha amplitude in the eyes-closed resting state might be more difficult to down-regulate alpha. Further efforts are needed to investigate both alpha up-regulation and down-regulation jointly.

Nevertheless, the correlation is only at a moderate level, it could not provide precise prediction of learner/non-learner. In order to predict whether a participant is a learner or not, a prediction model with more useful features are required. Therefore, we utilized a stepwise LDA to find out significant predictor variables and build a prediction model. As a result, the amplitudes in theta, lower alpha, sigma and beta 1 frequency band in the eyes-closed resting baseline before NF were identified as the significant predictor variables, suggesting the importance of eyes-closed resting EEG activities in prediction of alpha learning. The leave-one-out cross-validation accuracy of 86.2% indicated the high performance of the prediction model. To some extent, our result is consistent with previous findings that resting EEG activities predict the within-session learning in other NF protocols (Wan et al., 2014; Reichert et al., 2015).

The future work may include the following issues. First, we would investigate the learning in the alpha down-regulation NF on other populations, with up-regulation jointly, so as to summarize and compare all the results of both up and down directions among different populations to find commons and differences, and ultimately reveal the underlying mechanism of the alpha NF. Second, besides the EEG learning during NF training, it is interesting to see whether the learning effects could sustain in situations outside training sessions. No-feedback retention or transfer tests would be helpful to answer this question. Finally, it is ultimately important to explore the learning effects on functional changes.

Taken together, the learning to down-regulate alpha had large inter-individual difference, indicating the importance of analyzing learning individually for deep understanding the NF and validation of NF efficiency. Importantly, we found that the broad band EEG activities in the eyes-closed resting baseline before NF training could predict the learner/non-learner. From NF practical aspect, the findings can provide a simple and easy way to predict alpha learning, as it only needs two min of eyes-closed resting EEG recording at the training location. Moreover, it would be very helpful for non-learners to save time and provide a better basis for adapting the training protocol accordingly.

## Author Contributions

WN, FW, and AR designed the research. WN analyzed the data and wrote the manuscript. QT conducted the experiment and analyzed the data. CW designed the neurofeedback platform. BW was involved in data analysis. FW and AR supervised, revised, and gave the final approval of the manuscript. All authors read and approved the manuscript.

## Conflict of Interest Statement

The authors declare that the research was conducted in the absence of any commercial or financial relationships that could be construed as a potential conflict of interest.
